# *Morganella morganii* NM-11 of snake origin: drug resistance profile, pathogenic potential, and impact on host gut microbiota

**DOI:** 10.3389/fvets.2026.1871413

**Published:** 2026-06-25

**Authors:** Baitong Chen, Mengrui Li, Shengxuan Jia, Yang Yang, Zhanghang Chen, Lei Wang, Panpan Guo, Haiyan Wu, Yutong Yan, Dengfeng Wang, Guangwen Yin

**Affiliations:** 1 College of Animal Sciences, Fujian Agriculture and Forestry University, Fuzhou, China; 2 Fuzhou Zoo Administration, Fuzhou, China

**Keywords:** antibiotic resistance, gut microbiota, molecular identification, *Morganella morganii*, multidrug-resistant

## Abstract

To date, cases of snake infection caused by *Morganella morganii* and its effects on the intestinal microecology of snakes remain poorly documented. A pathogenic strain, designated NM-11, was isolated from diseased and dead snakes collected from a snake farm in China. In this study, strain NM-11 was identified as *Morganella morganii* through morphological observation, physiological and biochemical characterization, 16S rRNA gene sequencing, as well as genome-based average nucleotide identity (ANI) and digital DNA–DNA hybridization (dDDH) analyses. Subsequently, artificial challenge assays were performed to evaluate its pathogenicity. The whole genome of the strain was sequenced using PacBio long-read technology, and antimicrobial susceptibility tests were conducted to characterize its antibiotic resistance profile. Furthermore, high-throughput sequencing was applied to explore the alterations in intestinal microbial diversity of *Ptyas mucosus* upon infection with strain NM-11. Pathogenicity assays revealed that strain NM-11 exhibited strong virulence. After experimental infection, *Ptyas mucosus* displayed typical clinical signs including depression, lethargy and diarrhea. Gross pathological examination revealed extensive hemorrhage in the abdominal cavity, lungs and gastrointestinal tract, and histopathological observation confirmed severe lesions in multiple organs. Antimicrobial susceptibility tests confirmed the strain was multidrug-resistant (MDR). It had high resistance to many antibiotics. These drugs include polymyxin B, tetracycline, cefuroxime, cephalexin, erythromycin, vancomycin and streptomycin. Intestinal microbiota analysis indicated that infection with NM-11 remarkably reshaped the intestinal microbial community structure of *Ptyas mucosus* and increased the overall microbial diversity, accompanied by a significant rise in the relative abundance of *Morganella morganii*. This is the first report of *Morganella morganii* isolated and identified from diseased snakes in China. Our findings clarify the pathogenic characteristics and antibiotic resistance profile of this resistant strain against *Ptyas mucosus*. The results provide fundamental scientific evidence for the clinical diagnosis and antimicrobial prevention and treatment of *Morganella morganii* infection in snakes, and also offer valuable insights for reptile medicine and public health risk prevention and control.

## 1 Introduction

In the era of rapid social advancement and the vigorous development of the pet economy, people’s preferences for companion animals have become increasingly diversified. Snakes possess features such as high ornamental value, small space requirements for breeding, and ease of management, which have attracted a growing number of pet enthusiasts (1). Thus, it can be concluded that pet snakes have become an indispensable part of the Chinese pet market. In addition to serving as companion animals and providing emotional value to humans. Snakes, as special economic animals, also possess extremely high economic value, which is mainly reflected in their medicinal values (2). After processing and preparation, snakes are made into various patented traditional Chinese medicine preparations. For example, snake gall is one of the principal ingredients of Pien Tze Huang, a renowned traditional Chinese medicine (3); techniques such as the extraction of venom from live snakes, the preservation of its lyophilized powder, the preparation of antivenom serum (4), and the isolation of antithrombin from snake venom have transformed traditional snake-based medicinal substances into modern high-tech products (5).

*Morganella morganii* is a Gram-negative facultative anaerobic bacterium that exists extensively in the environment and the intestinal tracts of animals and is categorized as a conditional pathogen (6). In the snake breeding industry, *Morganella morganii* infection can lead to severe health issues and economic losses. Particularly prone to outbreak under circumstances of high-density breeding or poor environmental management. In the domain of research concerning *Morganella morganii*, its drug resistance has perpetually remained a major research emphasis (7). Research has reported *Morganella morganii* strains resistant to third-generation cephalosporins in dolphins (8). Additionally, researchers have successfully isolated different *Morganella morganii* strains from a series of infected patients. Through drug susceptibility testing, they found that each isolated strain exhibited resistance to at least three different antibiotics, providing compelling evidence of its multidrug-resistant characteristics (9). The pathogenicity of this bacterium extends to various animal species, causing skin ulcers and bleeding in aquatic animals (10), and symptoms such as diarrhea, lethargy, and respiratory diseases in amphibians (11) and mammals (12). In severe cases, it can even lead to animal death. Notably, case reports have also linked *Morganella morganii* to human brain abscesses (13), bacteremia (14), and sepsis (15).

The gut microbiota plays a critical role not only in maintaining host health but also in the pathogenesis of gastrointestinal disorders (16). Pathogenic infections may alter the host’s microbial community (17). Recent studies have highlighted the critical role of microbial communities in host health (18), particularly in immune function (19). Due to its unique structure, the gut is continuously exposed to diverse antigens and microorganisms (20). A bidirectional interaction exists between gut microbiota and the host, the nature of which is influenced by the composition and abundance of the microbial community (21).

This paper reports a highly contagious disease outbreak on a large *Ptyas mucosus* farm in Fujian Province, China. Infected snakes exhibited clinical symptoms including loose bloody diarrhea, open-mouth breathing and oral secretions, resulting in mortality of several snakes. The pathogen was identified as *Morganella morganii* through pathological autopsy, bacterial isolation and purification, combined with morphological, physiological and biochemical identification, 16S rRNA gene sequencing and whole-genome sequencing. Furthermore, we comprehensively analyzed the antibiotic resistance profile of the isolate via animal challenge tests, PacBio third-generation sequencing and antimicrobial susceptibility testing. Meanwhile, high-throughput sequencing was used to clarify the effects of *Morganella morganii* infection on intestinal microbial diversity in *Ptyas mucosus*.

Currently, research on *Morganella morganii* has predominantly focused on clinical isolates from humans (22) and strains derived from livestock and poultry (23). In contrast, systematic investigations into isolates from reptiles such as snakes remain scarce, with substantial knowledge gaps in areas including antibiotic resistance profiles, virulence factor distribution, and impacts on host intestinal microecology. As critical natural reservoirs of *Morganella morganii*, snakes may harbor strains that can be transmitted to humans via the food chain or direct contact, thereby presenting a potential public health risk (24). Consequently, systematic studies on snake-derived *Morganella morganii* hold significant scientific importance and practical value. This study intends to systematically analyze the pathogenicity, antibiotic resistance characteristics, and induced intestinal dysbiosis of a snake-origin *Morganella morganii* isolate.

## 2 Materials and methods

### 2.1 Animals

The naturally infected *Ptyas mucosus* and twelve 21-day-old healthy *Ptyas mucosus* used in this study were sourced from a commercial snake farm in Fujian Province, China. All animals were maintained under controlled environmental conditions: a constant temperature of 25 °C, relative humidity of 70%, and a 12-h light/dark cycle provided by a UVB light source (wavelength 290–320 nm). Clean drinking water was provided ad libitum throughout the study period. All animal procedures received prior approval from the Institutional Animal Care and Use Committee (IACUC) of Fujian Agriculture and Forestry University, Fuzhou, Fujian Province, China. The number of laboratory animal use license is SYXK 2025-0006.

### 2.2 Postmortem examination

Under sterile conditions, a necropsy was performed on the deceased snake. The subcutaneous tissue, muscles, and body cavities were meticulously examined for the presence of egg masses or parasitic infestations. Subsequently, the snake’s abdomen was dissected, and its organs were carefully separated in sequence. A thorough inspection was conducted to assess the coloration and the presence of abscesses on the surface of solid organs such as the lungs and liver. Additionally, the digestive tract was examined for any signs of abscesses and hemorrhage.

### 2.3 Bacteriologic samples

The NM-11 strain was obtained from a diseased snake during an outbreak investigation at a snake farm located in Fujian Province, China. After dissecting the diseased snake, sterile inoculating loops, previously sterilized by flaming, were utilized to puncture tissues such as the lungs, heart, and liver. Subsequently, streak cultures were performed on solid agar media, which were then sealed and inverted for incubation in a bacterial incubator at 30 °C. After 24 h, colony morphologies were observed and recorded (23). Dominant single colonies from each plate were selected and re-streaked onto fresh solid media for purification (25). This process was repeated three times to obtain pure strains. Select purified single bacterial colonies from the sterilized inoculation ring and inoculate them into LB liquid medium. Incubate on a shaker for 24 h (30 °C, 200r·min^−1^), then add 50% glycerol of the same volume and store in a −80 °C freezer to preserve the bacterial strains (26). The isolated and purified strains were selected for Gram staining and microscopy.

### 2.4 Physiological and biochemical tests

To test biochemical characteristics, the isolated and purified NM-11 strain was inoculated into a series of micro-biochemical reaction tubes (14 types, including phenylalanine deaminase, urease, and lysine decarboxylase). Each reaction type was set up in quadruplicate, with three replicates inoculated with the bacterium and one left uninoculated as a control. All bacterial micro-biochemical reaction tubes were purchased from Hangzhou Microbial Reagent Co., Ltd., Zhejiang Province, China, with catalog number J2002. Strictly follow the standards outlined in the Bergey’s Manual of Determinative Bacteriology and the corresponding biochemical identification kit instructions for interpretation. Biochemical results were determined based on medium color, turbidity and gas production, with observations conducted 24–48 h after inoculation. The reference strain *Morganella morganii* ATCC 25830 is a Gram-negative, facultative anaerobic bacillus. It is characterized by positive reactions for urease, indole and ornithine decarboxylase, ferments glucose with acid and gas production, and is unable to ferment lactose.

### 2.5 PCR and 16S rRNA gene identification

Genomic DNA was extracted using a DNA extraction kit (Yeasen Biotechnology Co., Ltd., Shanghai, China) following the manufacturer’s instructions. The extracted DNA served as the template for PCR amplification of the 16S rRNA gene using universal bacterial primers (27F: 5′-AGAGTTTGATCCTGGCTCAG-3′ and 1492R: 5′-TACGGCTACCTTGTTACGACTT-3′), synthesized by Fuzhou Biosune Biotechnology Co., Ltd. The PCR reaction mixture consisted of 12.5 μL of rTaq polymerase, 8.5 μL of ddH₂O, 1 μL each of forward and reverse primers, and 2 μL of DNA template. The amplification program included an initial denaturation at 94 °C for 5 min, followed by 30 cycles of denaturation at 94 °C for 30 s, annealing at 58 °C for 30 s, and extension at 72 °C for 1 min, with a final extension at 72 °C for 10 min, and held at 4 °C. The PCR products were sequenced by Sangon Biotech Co., Ltd. (Shanghai, China). The resulting sequences were subjected to BLAST analysis against the NCBI database, and a phylogenetic tree was constructed using MEGA version 7.0.

### 2.6 Antimicrobial susceptibility

Using the Kirby-Bauer method (27), sensitivity testing was conducted for the strain against 20 common antibiotics. All antimicrobial susceptibility test discs used were purchased from Changde Bickman Biotechnology Co., Ltd., Hunan Province, China, batch number: 20241021. A single colony of the purified strain was inoculated into LB liquid medium and incubated at 30 °C with shaking at 200 r·min^−1^ for 24 h prior to antimicrobial susceptibility testing. All procedures were performed in a laminar flow hood: 200 μL of the bacterial culture was evenly spread onto nutrient agar plates using a sterile spreader that had been flame-sterilized and cooled to room temperature. After the agar surface had dried, each plate was divided into four equal sections, and antimicrobial disks were gently placed at the center of each section using sterile forceps. The plates were sealed, inverted, and incubated at 30 °C for 24 h. The diameters of the inhibition zones (including the 6 mm disk) were measured using a vernier caliper. The results were interpreted according to the 2017 edition of the CLSI guidelines for antimicrobial susceptibility testing, CLSI2017-M100.

### 2.7 Whole genome sequencing and assembly

Genomic DNA of strain NM-11 was extracted and subjected to whole genome sequencing on the PacBio Sequel II platform by Wuhan Frasergen Bioinformatics Co., Ltd. Third-generation sequencing data were assembled and corrected using HGAP4 and Canu 1.6 software, and the subreads were aligned to the assembled genome with pbalign 0.4.1 to analyze coverage depth distribution. The HGAP algorithm leverages the long-read advantage of the PacBio platform to perform error correction, assembly, and optimization, ultimately yielding a high-quality genome assembly.

### 2.8 Gene annotation and analysis

Coding regions were identified using Glimmer (v3.02). tRNA genes and 5S, 16S, and 23S rRNA genes were predicted using tRNAscan-SE (v2.0) and RNAmmer, respectively, while other non-coding RNAs were predicted using Infernal (v1.1.2). Protein sequences were predicted via the blastp command in diamond and compared against the NCBI NR bacterial database, SwissProt, COG, KEGG, and Gene Ontology (GO) databases for functional annotation, with thresholds set at E ≤ 1 × 10^−5^ and sequence identity ≥60%. The predicted protein sequences were also aligned against the Comprehensive Antibiotic Resistance Database (CARD) for resistance gene annotation using the same criteria.

### 2.9 Animal challenge test

The pathogenicity of the NM-11 strain was evaluated by infecting 21-day-old fasted *Ptyas mucosus* with the isolated strain. To assess its pathogenic potential, the snakes were divided into four groups of three individuals each. The isolated and purified NM-11 strain was prepared into three concentration gradients: 5.4 × 10^9^ CFU/mL, 5.4 × 10^8^ CFU/mL, and 5.4 × 10^7^ CFU/mL. Each concentration was administered via intraperitoneal injection to a separate experimental group, while another group received an equal volume of saline solution as the control. Detailed information is provided in Table 1.

**Table 1:** *Ptyas mucosus* attack test dose.

Group	Number of animals	Injection concentration	Injection dose
Test group M1	3	5.4 × 10^9^ CUF/mL	0.6 mL
Test group M2	3	5.4 × 10^8^ CFU/mL	0.6 mL
Test group M3	3	5.4 × 10^7^ CFU/mL	0.6 mL
Control Group E	3	0.9% NaCl	0.6 mL

All groups were maintained under identical feeding conditions and provided only sterile water throughout the experiment. Following inoculation, the behavioral and physiological status of the snakes in each group was recorded every 6 h, and any mortality was promptly documented. Deceased snakes underwent immediate pathological dissection under sterile conditions for bacterial identification. Tissue samples from the heart, lungs, liver, stomach, and intestines were fixed in formalin for 24 h and processed for histopathological examination.

### 2.10 Characteristics of the gut microbiota in *Ptyas mucosus* infected with the NM-11 strain

To investigate the potential impact of the NM-11 strain on intestinal microbiota, we collected intestinal contents from experimental animals for 16S rRNA, followed by comprehensive bioinformatic analyses. Intestinal contents were aseptically collected immediately from *Ptyas mucosus* that died during the challenge test; samples from surviving individuals were collected uniformly at the end of the 14-day observation period. All experimental procedures were performed aseptically in a Class II biological safety cabinet. The study comprised three infected groups (M1–M3, *n* = 3 per group) and one control group (E, *n* = 3). Intestinal contents were collected from *Ptyas mucosus* specimens. The intestinal middle segment (1 cm excised from both ends) was opened to collect luminal contents into sterile cryotubes, which were then accurately weighed (0.1 mg precision) and flash-frozen. A total of 12 intestinal content samples were stored at −80 °C.

Whole genome sequencing targeting the 16S rRNA V3-V4 region was conducted using the Illumina MiSeq platform (Shanghai OE Biotech Co., Ltd.) with paired-end sequencing. After quality control and statistical processing of the raw sequencing data, bioinformatic analyses were performed, including OTU clustering, assessment of alpha and beta diversity, and characterization of the microbial community structure.

## 3 Results

### 3.1 Tissue dissection of diseased animal lesions

**Figure 1:**
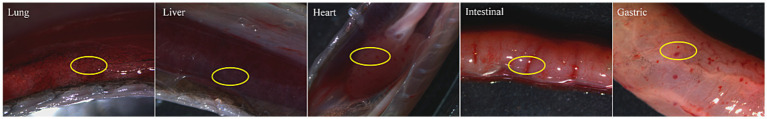
Necropsy of a diseased *Ptyas mucosus*.

Upon observation, the main symptoms of the diseased snakes included diarrhea with watery, blood-tinged feces, accompanied by open-mouth breathing. Their lungs exhibited visible congestion and mild enlargement (Figure 1), their livers were enlarged and dark red (Figure 1), their hearts were congested and showed hemorrhagic spots (Figure 1), their gastrointestinal tracts showed hemorrhagic spots (Figure 1).

### 3.2 Strain purification and gram staining

**Figure 2:**
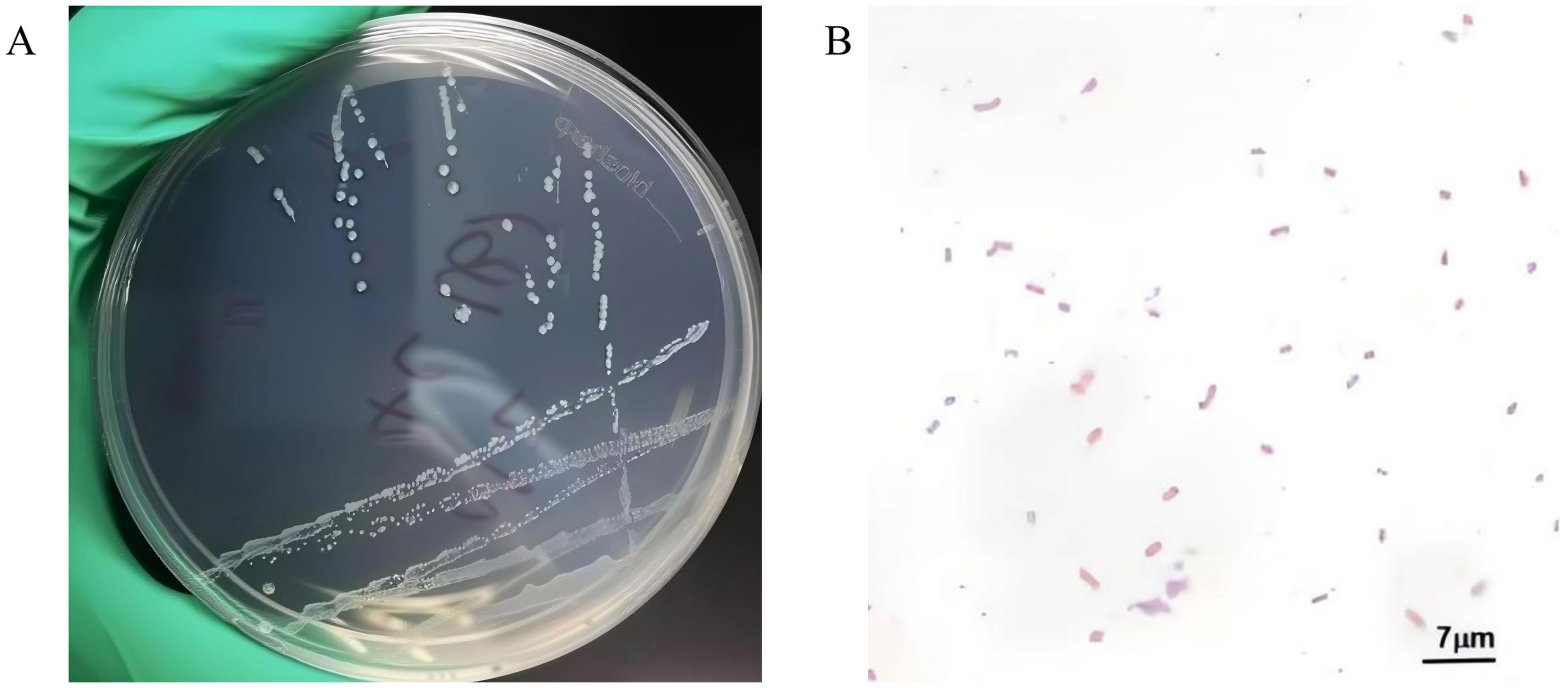
Isolation and characterization of the NM-11 bacterial strain. **(A)**: Colonial morphology of strain NM-11. **(B)**: Morphology of strain NM-11 under Gram staining.

Colonies with similar morphologies were isolated from the kidney, liver, and other tissues of the diseased snakes. Through plate purification, circular, smooth, white opaque colonies with neat edges were obtained on the plates (Figure 2A). The bacterial strain was assigned the designation NM-11. Gram staining revealed that the bacterial cells were short rod-shaped with blunt ends and stained red, indicating they were Gram-negative bacteria (Figure 2B).

### 3.3 Biochemical characterization

The biochemical profiles of strain NM-11 are listed in Table 2. The biochemical characteristics of the NM-11 strain were compared with the typical features of the *Morganella morganii* standard strain. Glucose fermentation was positive. The medium gradually turned red to yellow at 8 h post-inoculation, gas formation occurred at 12 h, and deep yellow staining with full gas filling in the Durham tube was observed at 24 h. Ornithine decarboxylase activity was positive, with the medium changing from yellow to pale purple at 12 h and uniform dark purple at 24 h. Urease reaction showed strongly positive results; the medium shifted from pale yellow to purplish-red within 6 h and became evenly dark purple at 24 h. After 24 h incubation, all fermentation media of lactose, maltose, sucrose, mannitol, sorbitol and inositol remained original red without color change, presenting negative results. The medium of lysine decarboxylase assay stayed yellow throughout the experiment with a negative outcome. No color shift was observed in phenylalanine deaminase medium after reagent addition, yielding negative result. Citrate utilization test showed negative response, with medium remaining green and no obvious bacterial growth. No red ring emerged on the liquid surface after Kovacs reagent addition in indole test, and the solution appeared pale yellow, which was judged negative. No black precipitate was detected in hydrogen sulfide production test, indicating negative result. Strain NM-11 shared nearly identical biochemical profiles with *Morganella morganii*, except for a negative indole reaction.

**Table 2:** Physiological and biochemical characteristics of the NM-11 strain.

Appraisal item	Results	Appraisal item	Results
Glucose	+	Lysine decarboxylase	−
Lactose	−	Ornithine decarboxylase	+
Maltose	−	Phenylalanine deaminase	−
Sucrose	−	Urease	+
Mannitol	−	Citrate	−
Euonymol	−	Indole	−
Inositol	−	H_2_S	−

“+” indicates positive; “-” indicates negative.

### 3.4 Molecular identification

**Figure 3:**
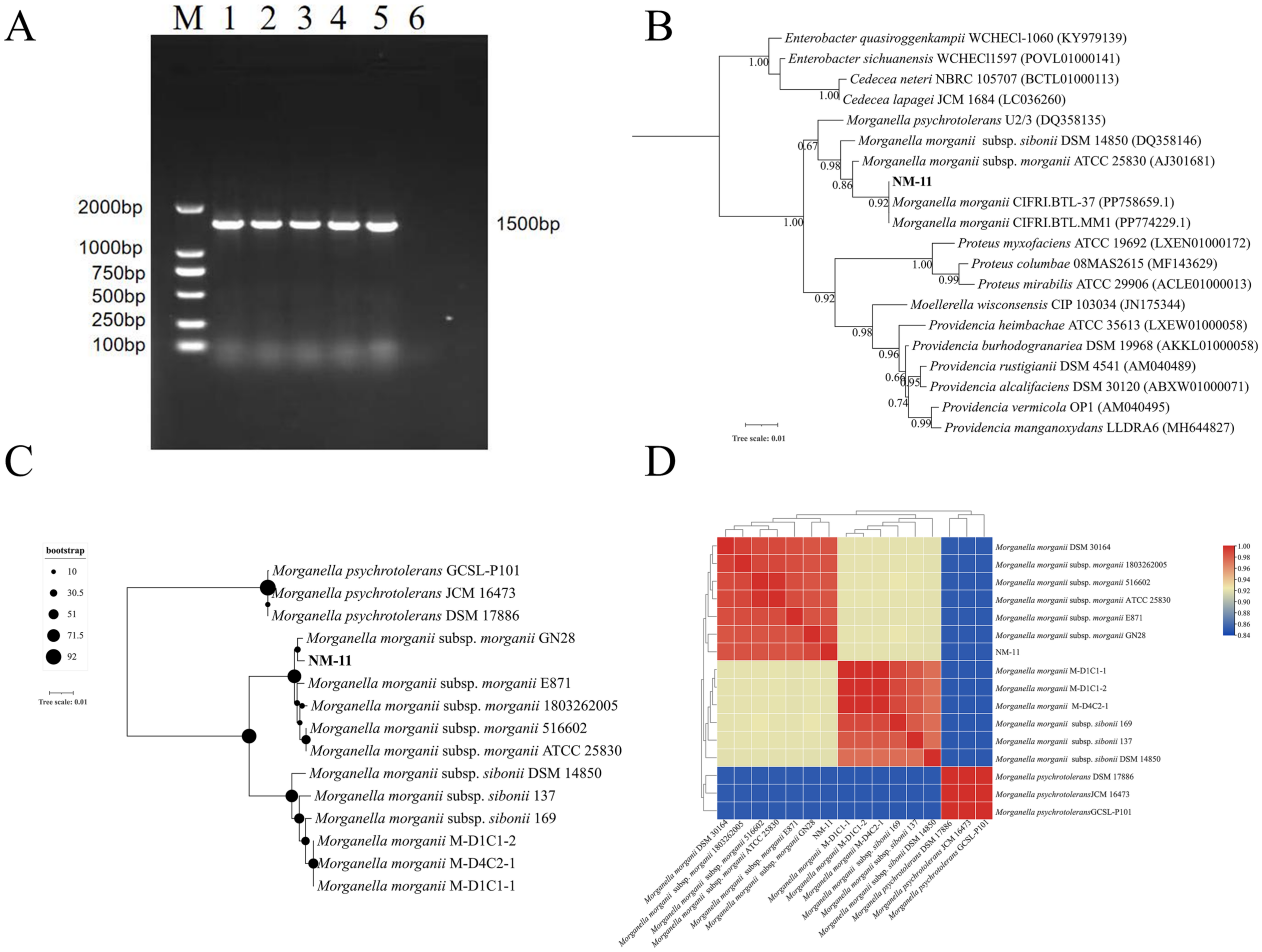
Figure showing molecular identification of the NM-11 strain. **(A)**: Gel electrophoresis result of the 16S rRNA gene amplification for the NM-11 strain, Note: M: Trans2000 DNA Marker, 1–5: 5 repetitions, 6: negative. **(B)**: Phylogenetic tree based on 16S rRNA gene sequence analysis of the NM-11 strain. **(C)**: Phylogenetic tree based on whole genome sequencing results of strain NM-11. **(D)**: ANI analysis between strain NM-11 and type strains of *Morganella* and related taxa.

The gel electrophoresis result of the NM-11 strain after 16S rRNA amplification is shown in the figure (Figure 3A). The band size is approximately 1,500 bp, and sequencing yielded a gene fragment with a full length of 1,439 bp (Supplementary Figure 1). NCBI BLAST analysis revealed that the 16S rRNA gene sequence of strain NM-11 shared >98.7% identity with those of *Morganella morganii*. A phylogenetic tree based on the 16S rRNA gene (Figure 3B) placed strain NM-11 in a clade with *Morganella morganii*, indicating the closest relationship to this species. Whole genome phylogenetic reconstruction (Figure 3C) and average nucleotide identity (ANI) analysis (Figure 3D) further demonstrated that strain NM-11 is most closely related to *Morganella morganii* subsp*. morganii* GN28. Digital DNA–DNA hybridization (dDDH) analysis yielded results consistent with these findings (Table 3), providing robust evidence for this taxonomic classification.

**Table 3:** Results of dDDH analysis between strain NM-11 and type strains of *Morganella* and related taxa.

Type strain	GenBank	dDDH
*Morganella morganii* subsp. morganii GN28	GCA_018802425.1	90.5%
*Morganella psychrotolerans* JCM 16473	GCA_039523655.1	25.9%
*Morganella morganii* subsp. sibonii DSM 14850	GCA_040560245.1	45%
*Morganella morganii* M-D1C1–2	GCA_044161715.1	45.7%

### 3.5 Drug sensitivity test results of the NM-11 strain

The test results (Table 4) indicated that the NM-11 strain was a multidrug-resistant strain: it was resistant to ampicillin, cefazolin, cefuroxime, cephalexin, erythromycin, lincomycin, vancomycin, penicillin, polymyxin B, streptomycin, and tetracycline; it showed moderate sensitivity to amikacin, cefoperazone, ceftazidime, doxycycline, gentamicin, kanamycin, and minocycline; and it was highly sensitive to ceftriaxone and piperacillin.

**Table 4:** Results of drug sensitivity test of 20 commonly used antibiotics.

Name	Drug	Contents/μg	Diameters of inhibitory zone/mm	Sensitivity
AMK	Amikacin	30	14.7	I
AMP	Ampicillin	10	6.0	R
CEZ	Cefazolin	30	11.1	R
CFO	Cefuroxime	30	6.1	R
CHA	Cefadroxil	30	6.6	R
CPZ	Cefoperazone	75	18.1	I
CTR	Ceftriaxone	30	23.3	S
CTZ	Ceftazidime	30	18.4	I
ERM	Erythromycin	15	6.1	R
GEN	Gentamicin	10	13.5	I
KAN	Kanamycin	30	16.3	I
LIN	Lincomycin	2	6.0	R
MIN	Minocycline	30	16.3	I
N30	Vancomycin	30	12.2	R
PEN	Penicillin	10	6.0	R
POL	Polymyxin B	300	6.0	R
PRL	Piperacillin	100	19.7	S
STM	Streptomycin	10	7.5	R
TET	Tetracycline	30	9.8	R
DOX	Doxycycline	30	15.9	I

S indicates highly sensitive, I indicates medium sensitivity, R indicates resistant.

### 3.6 Whole genome sequencing of *Morganella morganii*

**Figure 4:**
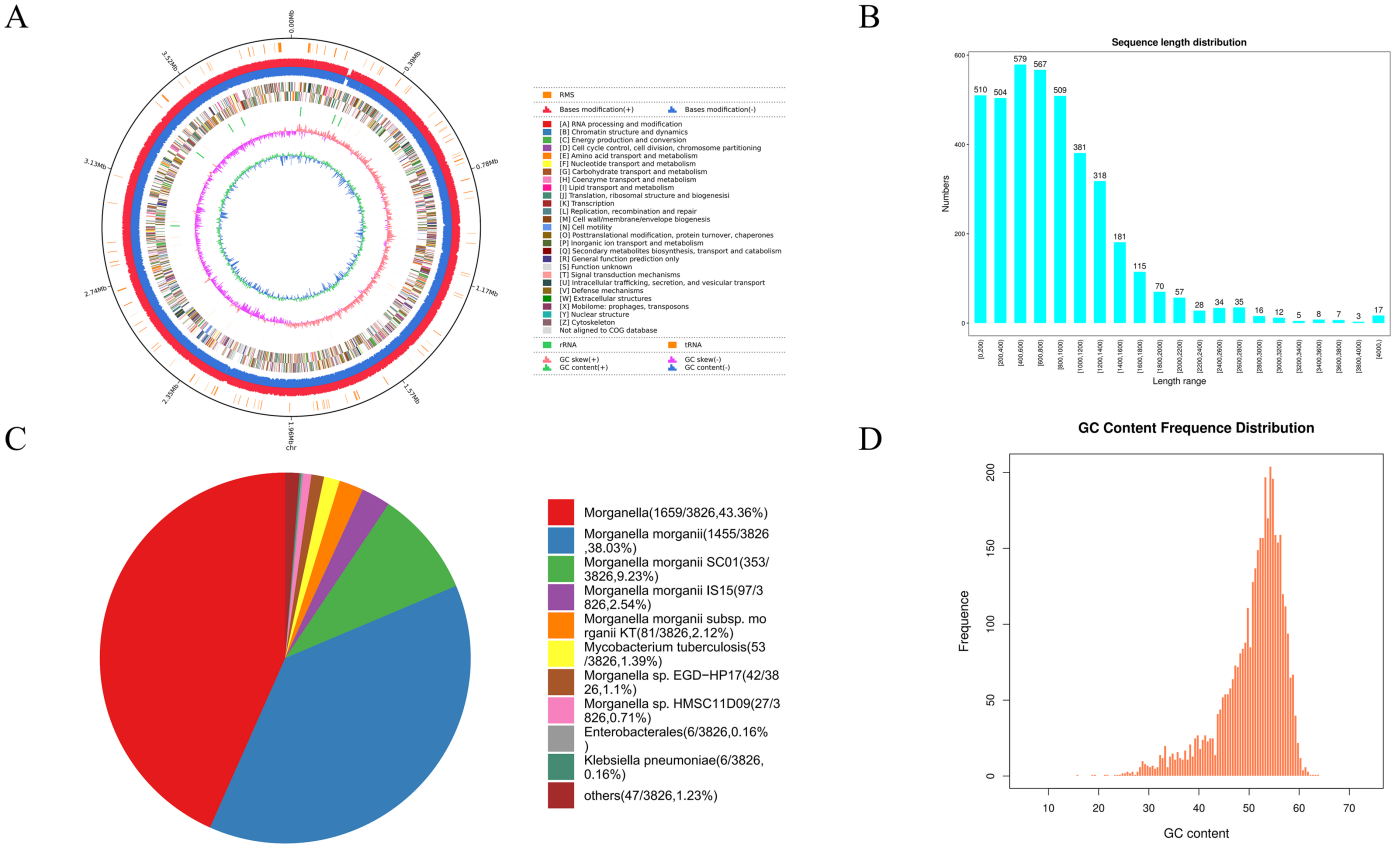
Whole genome sequencing map of strain NM-11. **(A)**: Circular genome map of strain NM-11. Note: The outermost circle indicates the chromosome genome size. The first inner circle shows restriction-modification sites, the second circle shows base modification sites, the third circle shows predicted CDS regions, the fourth circle shows non-coding RNA locations, the fifth circle shows GC skew analysis, and the innermost (sixth) circle shows GC content. **(B)**: Length distribution of protein-coding genes (CDS) predicted from the whole-genome sequencing data of strain NM-11. **(C)**: Species distribution of homologous sequences for NM-11 strain genes annotated against the NR database. **(D)**: GC content frequency distribution of the whole genome of strain NM-11.

Whole genome sequencing and assembly revealed that the genome of isolate NM-11 is a closed circular molecule of 3,914,747 bp, with a base composition of 24.44% A, 24.44% T, 25.55% C, and 25.58% G, corresponding to a GC content of 51.12%. A total of 3,956 protein-coding genes were predicted, with a cumulative coding sequence length of 3,428,529 bp, accounting for 87.58% of the genome. The average length of the coding genes was 866.67 bp, and their average GC content was 52.51% (Figures 4B,D). The genome also harbors 22 rRNA genes, 85 tRNA genes, and 60 other RNA elements. The rRNA genes include 7 copies of 16S rRNA, 8 copies of 5S rRNA, and 7 copies of 23S rRNA. The total length of tRNA genes is 6,671 bp, with an average length of 78.48 bp, representing 0.17% of the genome. The other RNAs fall into seven categories: antisense RNA, sRNA, antitoxin, riboswitch, ribozyme, leader, and thermoregulator, among which sRNA is the most abundant (20 copies), while ribozyme and antitoxin are the least abundant (one copy each). The lengths of these RNA types are summarized in Table 5. A circular genome map was generated based on the whole genome data (Figure 4A). A total of 3,938 genes of strain NM-11 were annotated against the NR database. Based on the annotation results, the 10 species with the highest matching rates were selected to generate a pie chart (Figure 4C). The two most abundant taxa were *Morganella* (43.36%) and *Morganella morganii* (38.03%), leading to the identification of the strain as *Morganella morganii*, which is consistent with the 16S rRNA gene-based identification. The gene sequence has been deposited in GenBank under accession number CP126137.

**Table 5:** Statistics of RNA prediction.

RNA type	Number	Total length/bp	Average length/bp	% of genome
tRNA	85	6,671	78.48	0.17%
5 s rRNA	8	920	115.00	0.02%
16 s rRNA	7	10,717	1,531.00	0.27%
23 s rRNA	7	20,344	2,906.29	0.52%
sRNA	20	2,454	112.70	0.06%
Antisense	3	369	123.00	0.01%
Antitoxin	1	149	149.00	0.01%
Leader	7	794	113.43	0.02%
Riboswitch	7	1,044	149.14	0.03%
Ribozyme	1	372	372.00	0.01%
Other type	5	1,875	152.35	0.05%

### 3.7 Drug resistance genes

The protein sequences of the predicted genes from strain NM-11 were aligned against the CARD database, resulting in the identification of 271 antimicrobial resistance genes (*E* value ≤ 1 × 10^−5^), accounting for 6.85% of all predicted genes. Among these, 22 genes exhibited sequence identity of ≥60% with known resistance genes. By antibiotic class (Table 6), the identified resistance genes included: 9 fluoroquinolone resistance genes (including two copies of *emrB*, *emrA*, *emrR*, *mdtH*, *acrB*, *Escherichia coli acrA*, *CRP*, *and H-NS*); 4 aminocoumarin resistance genes (*mdtC*, *mdtB*, *cpxA*, *baeR*); 4 cephalosporin resistance genes (*acrB*, *DHA-1*, *Escherichia coli acrA*, *H-NS*); 4 peptide antibiotic resistance genes (*bacA, PmrF, rosB, arnA*); 3 phenicol resistance genes (*catII*, *Escherichia coli acrA*, *acrB*); 3 aminoglycoside resistance genes (*kdpE*, cpxA, baeR); 3 tetracycline resistance genes (*acrB*, *Escherichia coli acrA*, *H-NS*); 2 cephamycin resistance genes (*DHA-1*, *H-NS*); 2 macrolide resistance genes (*CRP*, *H-NS*); 2 rifamycin resistance genes (acrB, *Escherichia coli acrA*); 2 glycylcycline resistance genes (*acrB*, *Escherichia coli acrA*); 1 nitroimidazole resistance gene (*msbA*); and 1 phosphonic acid resistance gene (*mdtG*). In addition, seven multidrug resistance genes were identified, namely *acrB*, *DHA-1*, *Escherichia coli acrA*, *CRP*, *baeR*, *cpxA*, *and H-NS*.

**Table 6:** Annotation results about drug resistance gene of CARD.

Drug class	Number of gene
Fluoroquinolone antibiotics	9
Aminocoumarin antibiotics	4
Cephalosporin antibiotics	4
Peptide antibiotics	4
Phenicol antibiotic	3
Aminoglycoside antibiotics	3
Tetracycline antibiotic	3
Cephamycin antibiotics	2
Macrolide antibiotics	2
Rifamycin antibiotics	2
Glycylcycline antibiotics	2
Nitroimidazole antibiotics	1
Phosphonic acid antibiotic	1

### 3.8 Results of animal challenge test

Pathogenicity assessment of the NM-11 strain in *Ptyas mucosus* revealed dose-dependent lethal effects. No mortality was observed within the first 6 h post-inoculation in any group. Snakes in the M1 (5.4 × 10^9^ CUF/mL) exhibited lethargy, reduced mobility, and delayed responsiveness beginning at 6 h. The first mortalities occurred in the M1 at 8 h, followed by complete mortality by 12 h. Concurrently, snakes in the M2 (5.4 × 10^8^ CFU/mL) displayed lethargy and diarrhea, with initial death at 12 h and full mortality by 24 h. In the M3 (5.4 × 10^7^ CFU/mL), the first death was recorded at 18 h. The remaining two snakes showed mild diarrhea and lethargy but retained moderate responsiveness. A second mortality occurred at 30 h, while the final surviving animal gradually recovered and remained alive through the 14-day observation period. Throughout the experimental period, all snakes in E showed no obvious abnormalities.

**Figure 5:**
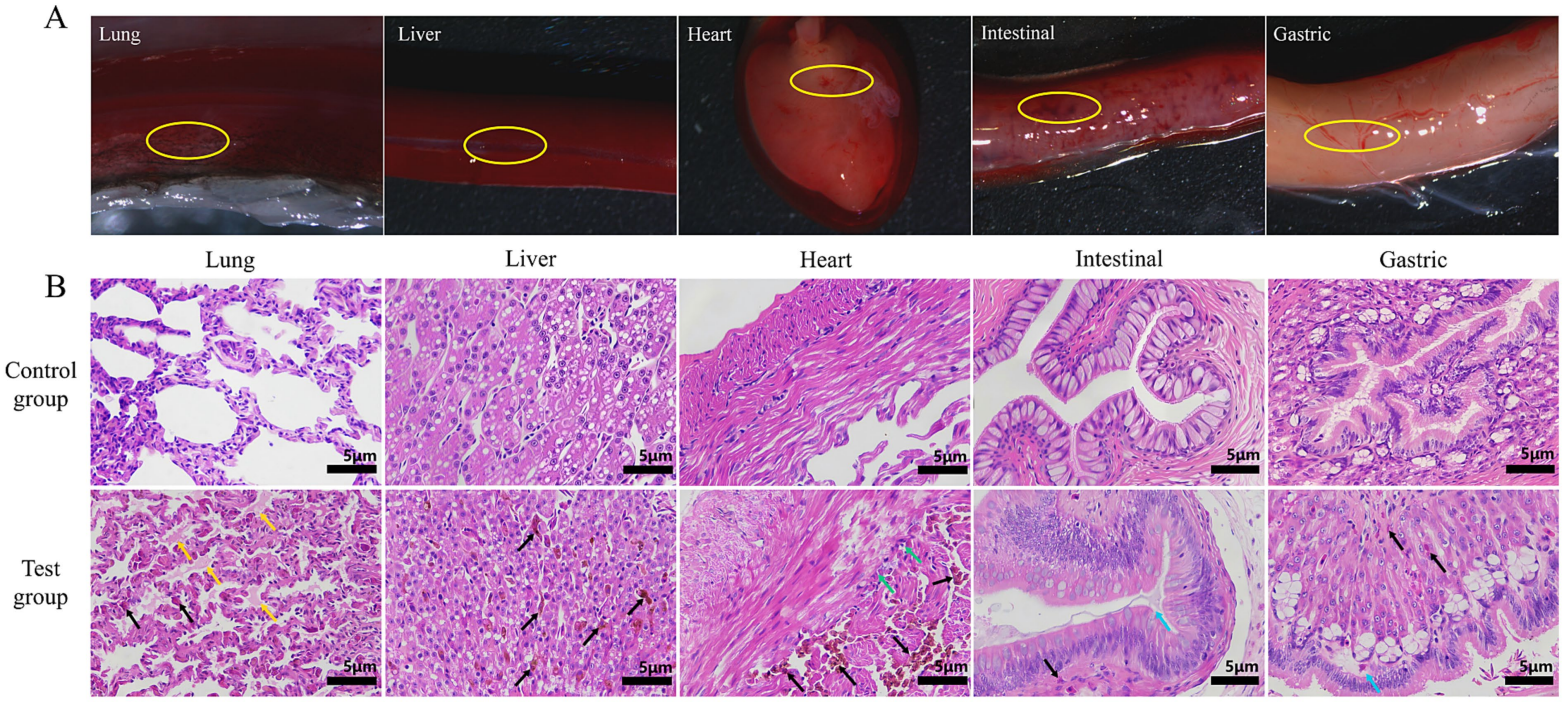
Pathological evaluation of *Ptyas mucosus* challenged with strain NM-11: Gross anatomical and histopathological examination. **(A)**: Pathological dissection of *Ptyas mucosus* after challenge with strain NM-11. **(B)**: Histopathological examination of *Ptyas mucosus* following challenge with strain NM-11 (H.E. stain 400x). Note: Black arrow: hemorrhagic focus; Blue arrow: mucosal denudation; Green arrow: inflammatory cell infiltration; Yellow arrow: alveolar serous exudation.

The necropsy results of the *Ptyas mucosus* that died from infection are shown in Figure 5. The presence of numerous blood streaks in the lungs indicated hemorrhage as well (Figure 5A), their livers were enlarged and dark red (Figure 5A), their hearts were congested and showed hemorrhagic spots (Figure 5A), with severe and dense hemorrhagic spots in the intestine (Figure 5A), and Multiple hemorrhagic spots were also found in the stomach (Figure 5A).

Upon observation of the histopathological sections, it was found that the lungs, heart, liver, and gastrointestinal tract exhibited varying degrees of pathological changes (Figure 5B). Compared to the control group, the lungs showed evident serous exudation and hemorrhage within the alveoli, with reduced and structurally incomplete alveolar septa. Multiple hemorrhagic points were observed in the liver tissue (Figure 5B). The myocardial fibers had multiple hemorrhagic spots accompanied by inflammatory cell infiltration (Figure 5B). Both the stomach and intestines exhibited partial mucosal shedding accompanied by hemorrhage (Figure 5B). The clinical signs and pathological findings in *Ptyas mucosus* from the animal reversion model were consistent with those observed in the primary infected animals from the snake farm.

### 3.9 Impact of the NM-11 strain infection on the gut microbiota in *Ptyas mucosus*

#### 3.9.1 Sequencing analysis results and OTU clustering

**Figure 6:**
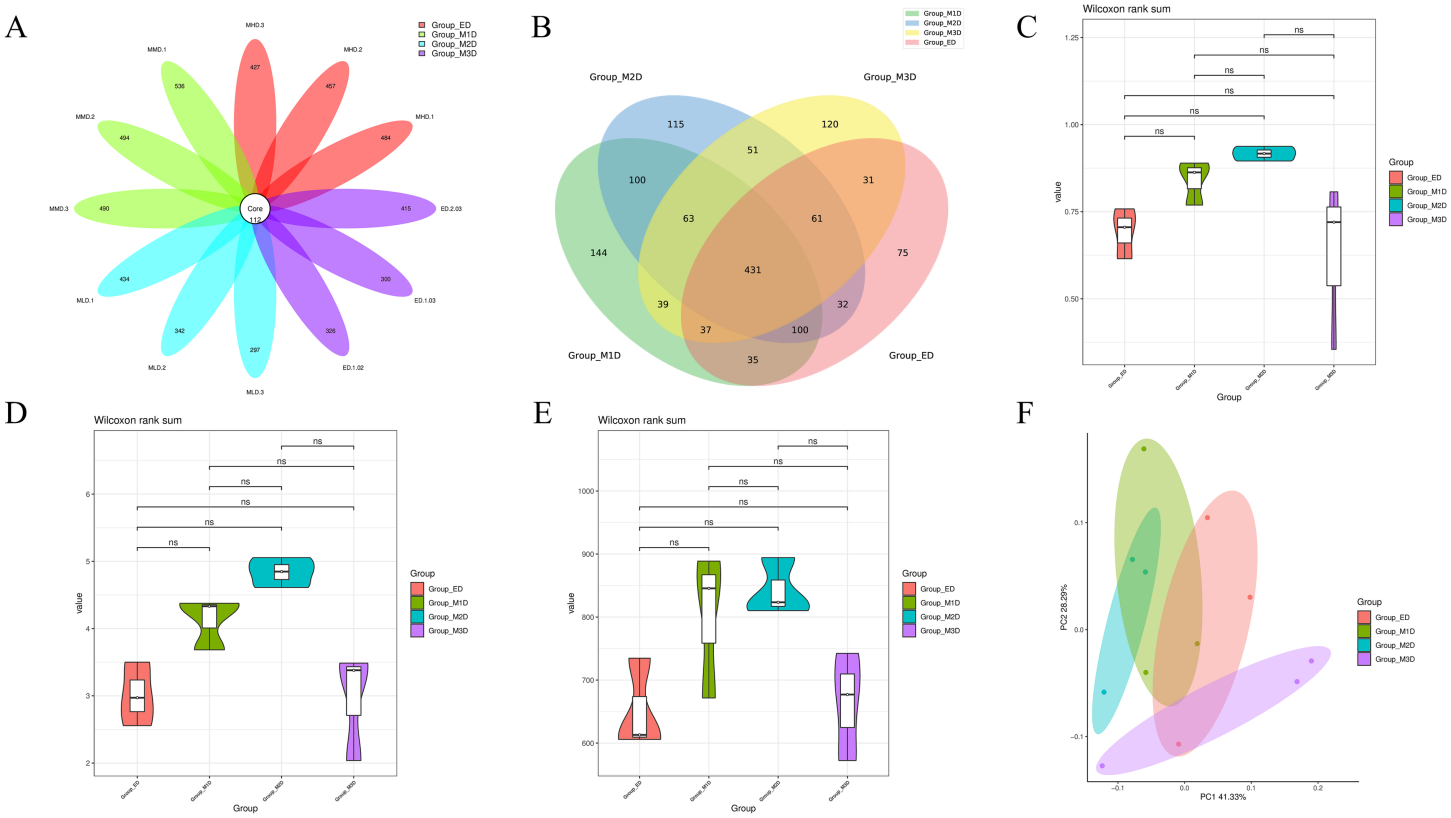
Impact of NM-11 strain infection on the gut microbiota diversity in *Ptyas mucosus.* **(A)**: Petal diagram for OTU clustering analysis of intestinal flora in *Ptyas mucosus*. **(B)**: Venn diagram of OTU clustering analysis for intestinal microbiota of *Ptyas mucosus*. **(C)**: Shannon index of alpha diversity analysis for intestinal microbiota of Ptyas mucosus. **(D)**: Simpson index of alpha diversity analysis for intestinal microbiota of Ptyas mucosus. **(E)**: Chao1 index Simpson index of alpha diversity analysis for intestinal microbiota of Ptyas mucosus. **(F)**: Beta diversity analysis of the gut microbiota in Ptyas mucosus. Note: M1 is the high-dose group (5.4 × 10^9^ CFU/mL); M2 is the medium-dose group (5.4 × 10^8^ CFU/mL); M3 is the low-dose group (5.4 × 10^7^ CFU/mL).

Based on the OTU clustering results of the intestinal microbiota, a petal diagram and Venn diagram were plotted. The petal diagram (Figure 6A) displaying OTU distribution across samples revealed that 112 OTUs were shared by all samples, which could be considered inherent to the intestinal microbiota of *Ptyas mucosus*. The inter-group Venn diagram (Figure 6B) showed that groups M1D, M2D, M3D, and ED contained 949, 953, 833, and 802 OTUs, respectively, with uniquely identified OTUs numbering 144, 125, 120, and 75 for each group. There were 431 OTUs common to all groups, while the numbers of OTUs shared between groups M1D, M2D, M3D, and ED were 603, 624, and 560, respectively.

#### 3.9.2 Alpha-diversity analysis results

In the alpha diversity analysis of the gut microbiota, analyses of the Shannon index (Figure 6C), Simpson index (Figure 6D), and Chao1 index (Figure 6E) collectively revealed that the M2D group exhibited the highest overall species diversity and the strongest reproducibility among replicates. In contrast, the M3D group showed lower diversity compared to the M2D group, along with poorer reproducibility. The M1D group displayed lower diversity than both the M2D and M3D groups, similar to group ED (control group). However, no statistically significant differences were observed among the four groups in the Chao1 index analysis (*p* > 0.05).

#### 3.9.3 Beta-diversity analysis results

Beta-diversity refers to the degree of diversity in biological communities and is commonly used to assess differences and correlations among sample groups (28). Principal coordinate analysis based on weighted Unifrac distance (Figure 6F) revealed that PC1 and PC2 explained 41.33 and 28.29% of the variation, respectively. Samples from each group (M1D, M2D, M3D) intermingled extensively, with high within-group dispersion, indicating no distinct group structure.

#### 3.9.4 Gut microbiota species richness

**Figure 7:**
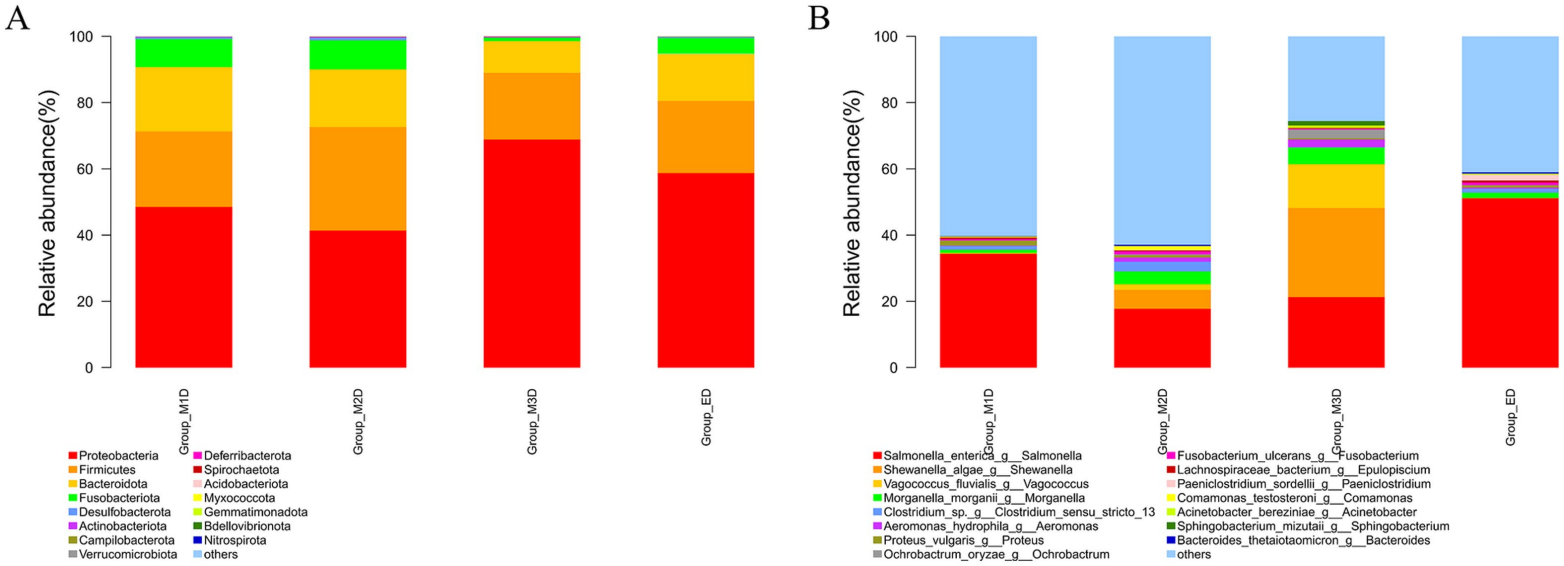
Impact of the NM-11 strain on gut microbiota abundance in *Ptyas mucosus*. **(A)**: Abundance of the top 15 species at phylum level in intestinal microbiota of *Ptyas mucosus*. **(B)**: Abundance of the top 15 species at the species level in the intestinal microbiota of *Ptyas mucosus*. Note: M1 is the high-dose group (5.4 × 10^9^ CFU/mL); M2 is the medium-dose group (5.4 × 10^8^ CFU/mL); M3 is the low-dose group (5.4 × 10^7^ CFU/mL).

In the intestinal content samples, the bacterial community structure showed no significant differences among groups at the phylum level (Figure 7A), with *Proteobacteria*, *Firmicutes*, and *Bacteroidetes* being the dominant phyla. At the species level (Figure 7B), however, differences were more pronounced, with relatively high abundance observed for *Salmonella enterica*, *Shewanella algae*, *Vagococcus fluvialis*, and *Morganella morganii*. Among these, the proportion of *Morganella morganii* was higher in the experimental groups compared to the control group, and within the experimental groups, it followed the order M3D > M2D > M1D.

## 4 Discussion

*Morganella morganii*, a facultative anaerobic and Gram-negative bacterium, is ubiquitous in nature (29), functioning both as an opportunistic pathogen and a zoonotic agent (30). Its pathogenic potential has been documented in various animal species, including dolphins (8), birds (23), and pigs (31). Notably, *Morganella morganii* accounts for a substantial proportion, up to 32%, of identified pathogens in snakebite-related infections (32). Despite this, there is a dearth of research on the pathogenic analysis of snake-derived *Morganella morganii* strains.

This study reports that *Morganella morganii* NM-11 poses a severe threat to snake health. Animal challenge experiments confirmed the pathogenicity of *Morganella morganii* NM-11, revealing a dose-dependent relationship between bacterial load and disease severity (33). The target strain was successfully re-isolated from dead individuals in the infection group, while no *Morganella morganii* NM-11 was detected in the control group. This result complies with Koch’s postulates and further verifies the pathogenicity of strain NM-11 (34). Histopathological analysis of the M1 group demonstrated partial detachment of the gastrointestinal mucosa. Gut microbiota analysis revealed that *Morganella morganii* NM-11 can alter the gut microbiome structure of *Ptyas mucosus* to some extent.

This study analyzed the antibiotic resistance of *Morganella morganii* strain NM-11. Antimicrobial susceptibility testing revealed that this strain exhibited a multidrug-resistant phenotype, consistent with previous findings (35). Annotation of the predicted gene protein sequences using the CARD database identified numerous resistance genes to various antibiotics, including fluoroquinolones, cephalosporins, peptide antibiotics, tetracyclines, aminoglycosides, chloramphenicols, and macrolides, similar to previous findings (36). Notably, resistance genes for cephalosporins, aminocoumarins, rifamycins and aminoimidazoles were identified in the genome. However, phenotypic susceptibility tests showed that the strain was only moderately resistant to these antibiotics, indicating a discrepancy between genotype and phenotype. Several potential reasons are proposed as follows: on one hand, many resistance genes are not constitutively highly expressed but are tightly regulated by promoter strength (37), induction conditions (38), and post-transcriptional controls (39), so low-level gene expression often confers only partial resistance; Moreover, temporary silencing of resistance genes may also be one of the reasons for the discrepancy between resistance genotypes and phenotypes (40); on the other hand, detected resistance genes might have functional defects such as gene truncation (41), pseudogenization (42), or encoding low-activity variants (43), rendering them incapable of full resistance. Notably, most resistance mechanisms require coordinated action among multiple genes to achieve complete resistance (44). Overall, the key point is that the presence of resistance genes is merely a necessary condition, not a sufficient one (45), for the development of resistance phenotypes, whose final expression is finely tuned by complex regulatory networks (46). These findings provide valuable insights for treating diseases caused by this bacterium in snake farms.

Gut microbiota analysis demonstrated that infection with *Morganella morganii* NM-11 increased microbial diversity in *Ptyas mucosus*. This shift may result from the reshaped interspecific competition following strain colonization (47). Notably, microbial diversity was lower in the high-dose group. Its diversity was also lower than that in the medium-dose and low-dose groups. This finding suggests gut microbiota diversity responds nonlinearly to pathogen load (48). Meanwhile, the composition of native gut microbiota changed. Colonization resistance also decreased (49). However, there were no significant differences in microbial abundance and community composition among the different dosage groups. This may be due to the inherent stability of the gut microbiota, as well as confounding factors such as host physiology, housing conditions, and individual variations (50). Overall, although *Morganella morganii* NM-11 infection did not induce directional changes in microbial composition, it was still associated with shifts in the gut microbial community structure of the *Ptyas mucosus*.

## 5 Conclusion

In this study, a pathogenic bacterial strain, designated NM-11, was isolated from an infected host. The strain NM-11 was identified as *Morganella morganii* based on morphological observations, physiological and biochemical characteristic analyses, 16S rRNA gene sequencing, and ANI and dDDH analyses derived from whole-genome sequencing. To further investigate its pathogenicity, an animal challenge experiment was conducted. Additionally, PacBio third-generation sequencing and antimicrobial susceptibility testing were employed to evaluate the resistance profile of the *Morganella morganii* NM-11 isolate. The results revealed that the strain exhibited multidrug resistance (MDR), showing significant resistance to multiple antibiotics. Analysis of microbial diversity in infected animals indicated that *Morganella morganii* NM-11 infection resulted in significant changes to the gut microbiota’s structure and abundance in *Ptyas mucosus*, along with effects on a range of associated bacterial functions. Notably, this study represents the first isolation of *Morganella morganii* from a snake host in China, providing crucial insights for future research on *Morganella morganii* infections in reptiles.

## Data Availability

The updated content reads: The data presented in the study are deposited in the GenBank repository, accession number CP126137.

## Supplementary material

SUPPLEMENTARY FIGURE 1 16S rRNA gene sequence obtained from strain NM-11 after amplification.

## References

[1] Valdez JW. Using Google trends to determine current, past, and future trends in the reptile pet trade. Animals (Basel). (2021) 11:676. doi: 10.3390/ani11030676, 33802560 PMC8001315

[2] Wang X Lin Y-Y Zhong W-T Ma Z-G Wu M-H Cao H . Evaluation of nutritional value of three kinds of medicinal snakes based on content of 15 amino acids. Zhongguo Zhong Yao Za Zhi. (2025) 50:2411–21. doi: 10.19540/j.cnki.cjcmm.20250216.104 40461197

[3] Chen Z. Pien Tze Huang (PZH) as a multifunction medicinal agent in traditional Chinese medicine (TCM): a review on cellular, molecular and physiological mechanisms. Cancer Cell Int. (2021) 21:146. doi: 10.1186/s12935-021-01785-3, 33658028 PMC7931540

[4] Tan CH. Snake venomics: fundamentals, recent updates, and a look to the next decade. Toxins (Basel). (2022) 14:247. doi: 10.3390/toxins14040247, 35448856 PMC9028316

[5] Oliveira AL Viegas MF da Silva SL Soares AM Ramos MJ Fernandes PA. The chemistry of snake venom and its medicinal potential. Nat Rev Chem. (2022) 6:451–69. doi: 10.1038/s41570-022-00393-7, 35702592 PMC9185726

[6] Jalandra R Dalal N Mohan A Solanki PR Kumar A. A novel method for enrichment of *Morganella morganii* in fecal samples using designed culture medium. Cell Biochem Funct. (2024) 42:e4004. doi: 10.1002/cbf.4004, 38583079

[7] Luo XW Liu PY Miao QQ Han RJ Wu H Liu JH . Multidrug resistance genes carried by a novel transposon Tn7376 and a Genomic Island named MMGI-4 in a pathogenic *Morganella morganii* isolate. Microbiol Spectrum. (2022) 10:e00265–22. doi: 10.1128/spectrum.00265-22, 35510850 PMC9241818

[8] Park SY Lee K Cho Y Lim SR Kwon H Han JE . Emergence of third-generation cephalosporin-resistant *Morganella morganii* in a captive breeding dolphin in South Korea. Animals (Basel). (2020) 10:2052. doi: 10.3390/ani10112052, 33171912 PMC7694518

[9] Al-Muhanna AS Al-Muhanna S Alzuhairi MA. Molecular investigation of extended-spectrum beta-lactamase genes and potential drug resistance in clinical isolates of *Morganella morganii*. Ann Saudi Med. (2016) 36:223–8. doi: 10.5144/0256-4947.2016.223, 27236395 PMC6074545

[10] Frith A Hayes-Mims M Carmichael R Björnsdóttir-Butler K. Effects of environmental and water quality variables on histamine-producing Bacteria concentration and species in the northern Gulf of Mexico. Microbiol Spectrum. (2023) 11:e0472022. doi: 10.1128/spectrum.04720-22, 37310253 PMC10434188

[11] Fernandes M Grilo ML Carneiro C Cunha E Tavares L Patino-Martinez J . Antibiotic resistance and virulence profiles of gram-negative bacteria isolated from loggerhead sea turtles (*Caretta caretta*) of the island of Maio, Cape Verde. Antibiotics (Basel). (2021) 10:771. doi: 10.3390/antibiotics10070771, 34202799 PMC8300689

[12] Liu H Zhu J Hu Q Rao X. *Morganella morganii*, a non-negligent opportunistic pathogen. Int J Infect Dis. (2016) 50:10–7. doi: 10.1016/j.ijid.2016.07.006, 27421818

[13] Elmi SM Obame FLO Dokponou YCH Yassin MR Attari SE El Asri ACC . Brain abscess caused by *Morganella morganii*: a case report and review of the literature. Surg Neurol Int. (2024) 15:7. doi: 10.25259/sni_759_2023, 38344080 PMC10858772

[14] Laupland KB Paterson DL Edwards F Stewart AG Harris PNA. *Morganella morganii*, an emerging cause of bloodstream infections. Microbiol Spectrum. (2022) 10:e0056922. doi: 10.1128/spectrum.00569-22, 35467403 PMC9241912

[15] Gameiro I Botelho T Martins AI Henriques R Lapa P. *Morganella morganii*: a rare cause of early-onset neonatal Sepsis. Cureus. (2023) 15:e45600. doi: 10.7759/cureus.45600, 37868540 PMC10588523

[16] Mohamed AS Bhuju R Martinez E Basta M Deyab A Mansour C . The gut microbiome's impact on the pathogenesis and treatment of gastric cancer-an updated literature review. Cancers (Basel). (2025) 17:2795. doi: 10.3390/cancers17172795, 40940892 PMC12427428

[17] Groves HT Cuthbertson L James P Moffatt MF Cox MJ Tregoning JS. Respiratory disease following viral lung infection alters the murine gut microbiota. Front Immunol. (2018) 9:182. doi: 10.3389/fimmu.2018.00182, 29483910 PMC5816042

[18] O'Riordan KJ Moloney GM Keane L Clarke G Cryan JF. The gut microbiota-immune-brain axis: therapeutic implications. Cell Rep Med. (2025) 6:101982. doi: 10.1016/j.xcrm.2025.101982, 40054458 PMC11970326

[19] Schluter J Peled JU Taylor BP Markey KA Smith M Taur Y . The gut microbiota is associated with immune cell dynamics in humans. Nature. (2020) 588:303–7. doi: 10.1038/s41586-020-2971-8, 33239790 PMC7725892

[20] Li T Han L Ma S Lin W Ba X Yan J . Interaction of gut microbiota with the tumor microenvironment: a new strategy for antitumor treatment and traditional Chinese medicine in colorectal cancer. Front Mol Biosci. (2023) 10:1140325. doi: 10.3389/fmolb.2023.1140325, 36950522 PMC10025541

[21] Socała K Doboszewska U Szopa A Serefko A Włodarczyk M Zielińska A . The role of microbiota-gut-brain axis in neuropsychiatric and neurological disorders. Pharmacol Res. (2021) 172:105840. doi: 10.1016/j.phrs.2021.105840, 34450312

[22] Mbelle N Osei Sekyere J Feldman C Maningi NE Modipane L Essack SY. Genomic analysis of two drug-resistant clinical *Morganella morganii* strains isolated from UTI patients in Pretoria, South Africa. Lett Appl Microbiol. (2020) 70:21–8. doi: 10.1111/lam.13237, 31630429

[23] Palmieri N Hess C Hess M Alispahic M. Sequencing of five poultry strains elucidates phylogenetic relationships and divergence in virulence genes in *Morganella morganii*. BMC Genomics. (2020) 21:579. doi: 10.1186/s12864-020-07001-2, 32831012 PMC7446228

[24] Peng Z Li X Hu Z Li Z Lv Y Lei M . Characteristics of carbapenem-resistant and colistin-resistant *Escherichia coli* co-producing NDM-1 and MCR-1 from pig farms in China. Microorganisms. (2019) 7:7. doi: 10.3390/microorganisms7110482, 31652858 PMC6920953

[25] Xie Q Yang M Duanmu Q Kang M Wang J Tan BE. Ningxiang pig-derived *Lactobacillus reuteri* improves the gut health of weaned piglets by regulating intestinal barrier function and cytokine profiles. Sci Rep. (2025) 15:3993. doi: 10.1038/s41598-025-87105-5, 39893246 PMC11787358

[26] Zhu J Ding J Yang K Zhou H Yang W Qin C . Microbiome and microbial pure culture study reveal commensal microorganisms alleviate *Salmonella enterica* serovar pullorum infection in chickens. Microorganisms. (2024) 12:1743. doi: 10.3390/microorganisms12091743, 39338418 PMC11434425

[27] Yang X Wang D Zhou Q Nie F Du H Pang X . Antimicrobial susceptibility testing of Enterobacteriaceae: determination of disk content and Kirby-Bauer breakpoint for ceftazidime/avibactam. BMC Microbiol. (2019) 19:240. doi: 10.1186/s12866-019-1613-5, 31675928 PMC6824082

[28] Wu Y Peng X Li X Li D Tan Z Yu R. Sex hormones influence the intestinal microbiota composition in mice. Front Microbiol. (2022) 13:964847. doi: 10.3389/fmicb.2022.964847, 36386696 PMC9659915

[29] O'Hara CM Brenner FW Miller JM. Classification, identification, and clinical significance of Proteus, Providencia, and Morganella. Clin Microbiol Rev. (2000) 13:534–46. doi: 10.1128/cmr.13.4.534, 11023955 PMC88947

[30] Chen J Wu Y Zhang G Kang W Wang T Li J . Tracing the possible evolutionary trends of *Morganella morganii*: insights from molecular epidemiology and phylogenetic analysis. mSystems. (2024) 9:e0030624. doi: 10.1128/msystems.00306-24, 38884495 PMC11264931

[31] Luo X Zhai Y He D Cui X Yang Y Yuan L . Molecular characterization of a novel Bla (CTX-M-3)-carrying Tn6741 transposon in *Morganella morganii* isolated from swine. J Med Microbiol. (2020) 69:1089–94. doi: 10.1099/jmm.0.001235, 32692646

[32] Bonilla-Aldana DK Bonilla-Aldana JL Ulloque-Badaracco JR Al-Kassab-Córdova A Hernandez-Bustamante EA Alarcon-Braga EA . Snakebite-associated infections: a systematic review and meta-analysis. Am J Trop Med Hyg. (2024) 110:874–86. doi: 10.4269/ajtmh.23-0278, 38507793 PMC11066359

[33] Chaichana P Satapoomin N Kullapanich C Chuenklin S Mohammad A Inthawong M . Comparative virulence analysis of seven diverse strains of *Orientia tsutsugamushi* reveals a multifaceted and complex interplay of virulence factors responsible for disease. PLoS Pathog. (2025) 21:e1012833. doi: 10.1371/journal.ppat.1012833, 40587585 PMC12237263

[34] Vouga M Greub G. Emerging bacterial pathogens: the past and beyond. Clin Microbiol Infect. (2016) 22:12–21. doi: 10.1016/j.cmi.2015.10.010, 26493844 PMC7128729

[35] Zhu W Liu Q Liu J Wang Y Shen H Wei M . Genomic epidemiology and antimicrobial resistance of Morganella clinical isolates between 2016 and 2023. Front Cell Infect Microbiol. (2024) 14:1464736. doi: 10.3389/fcimb.2024.1464736, 39958990 PMC11826060

[36] Behera DU Dixit S Gaur M Mishra R Sahoo RK Sahoo M . Sequencing and characterization of *M. morganii* strain UM869: a comprehensive comparative genomic analysis of virulence, antibiotic resistance, and functional pathways. Genes (Basel). (2023) 14:1279. doi: 10.3390/genes14061279, 37372459 PMC10298637

[37] Westmann CA Goldbach L Wagner A. The highly rugged yet navigable regulatory landscape of the bacterial transcription factor TetR. Nat Commun. (2024) 15:10745. doi: 10.1038/s41467-024-54723-y, 39737967 PMC11686294

[38] Nielsen TK Browne PD Hansen LH. Antibiotic resistance genes are differentially mobilized according to resistance mechanism. Gigascience. (2022) 11:11. doi: 10.1093/gigascience/giac072, 35906888 PMC9338424

[39] Galán JC González-Candelas F Rolain JM Cantón R. Antibiotics as selectors and accelerators of diversity in the mechanisms of resistance: from the resistome to genetic plasticity in the β-lactamases world. Front Microbiol. (2013) 4:9. doi: 10.3389/fmicb.2013.00009, 23404545 PMC3567504

[40] Kime L Randall CP Banda FI Coll F Wright J Richardson J . Transient silencing of antibiotic resistance by mutation represents a significant potential source of unanticipated therapeutic failure. MBio. (2019) 10:e01755–19. doi: 10.1128/mBio.01755-19, 31662453 PMC6819657

[41] Hall MB Lima L Coin LJM Iqbal Z. Drug resistance prediction for *Mycobacterium tuberculosis* with reference graphs. Microb Genom. (2023) 9:mgen001081. doi: 10.1099/mgen.0.001081, 37552534 PMC10483414

[42] Riffaud C Pinel-Marie ML Pascreau G Felden B. Functionality and cross-regulation of the four SprG/SprF type I toxin-antitoxin systems in *Staphylococcus aureus*. Nucleic Acids Res. (2019) 47:1740–58. doi: 10.1093/nar/gky1256, 30551143 PMC6393307

[43] Noel HR Petrey JR Palmer LD. Mobile genetic elements in Acinetobacter antibiotic-resistance acquisition and dissemination. Ann N Y Acad Sci. (2022) 1518:166–82. doi: 10.1111/nyas.14918, 36316792 PMC9771954

[44] Chiș AA Rus LL Morgovan C Arseniu AM Frum A Vonica-Țincu AL . Microbial resistance to antibiotics and effective Antibiotherapy. Biomedicine. (2022) 10:10. doi: 10.3390/biomedicines10051121, 35625857 PMC9138529

[45] Pesesky MW Hussain T Wallace M Patel S Andleeb S Burnham CD . Evaluation of machine learning and rules-based approaches for predicting antimicrobial resistance profiles in gram-negative Bacilli from whole genome sequence data. Front Microbiol. (2016) 7:1887. doi: 10.3389/fmicb.2016.01887, 27965630 PMC5124574

[46] Corona F Martinez JL. Phenotypic resistance to antibiotics. Antibiotics (Basel). (2013) 2:237–55. doi: 10.3390/antibiotics2020237, 27029301 PMC4790337

[47] Coyte KZ Schluter J Foster KR. The ecology of the microbiome: networks, competition, and stability. Science. (2015) 350:663–6. doi: 10.1126/science.aad2602, 26542567

[48] Segura Munoz RR Mantz S Martínez I Li F Schmaltz RJ Pudlo NA . Experimental evaluation of ecological principles to understand and modulate the outcome of bacterial strain competition in gut microbiomes. ISME J. (2022) 16:1594–604. doi: 10.1038/s41396-022-01208-9, 35210551 PMC9122919

[49] Bäckhed F Fraser CM Ringel Y Sanders ME Sartor RB Sherman PM . Defining a healthy human gut microbiome: current concepts, future directions, and clinical applications. Cell Host Microbe. (2012) 12:611–22. doi: 10.1016/j.chom.2012.10.012, 23159051

[50] Khan I Bai Y Zha L Ullah N Ullah H Shah SRH . Mechanism of the gut microbiota colonization resistance and enteric pathogen infection. Front Cell Infect Microbiol. (2021) 11:716299. doi: 10.3389/fcimb.2021.716299, 35004340 PMC8733563

