# Supplementary figures and images for: Morganella morganii NM-11 of snake origin: drug resistance profile, pathogenic potential, and impact on host gut microbiota
Source: Front Vet Sci. 2026 Jun 25;13:1871413. doi: 10.3389/fvets.2026.1871413 (PMC13345872; doi:10.3389/fvets.2026.1871413)

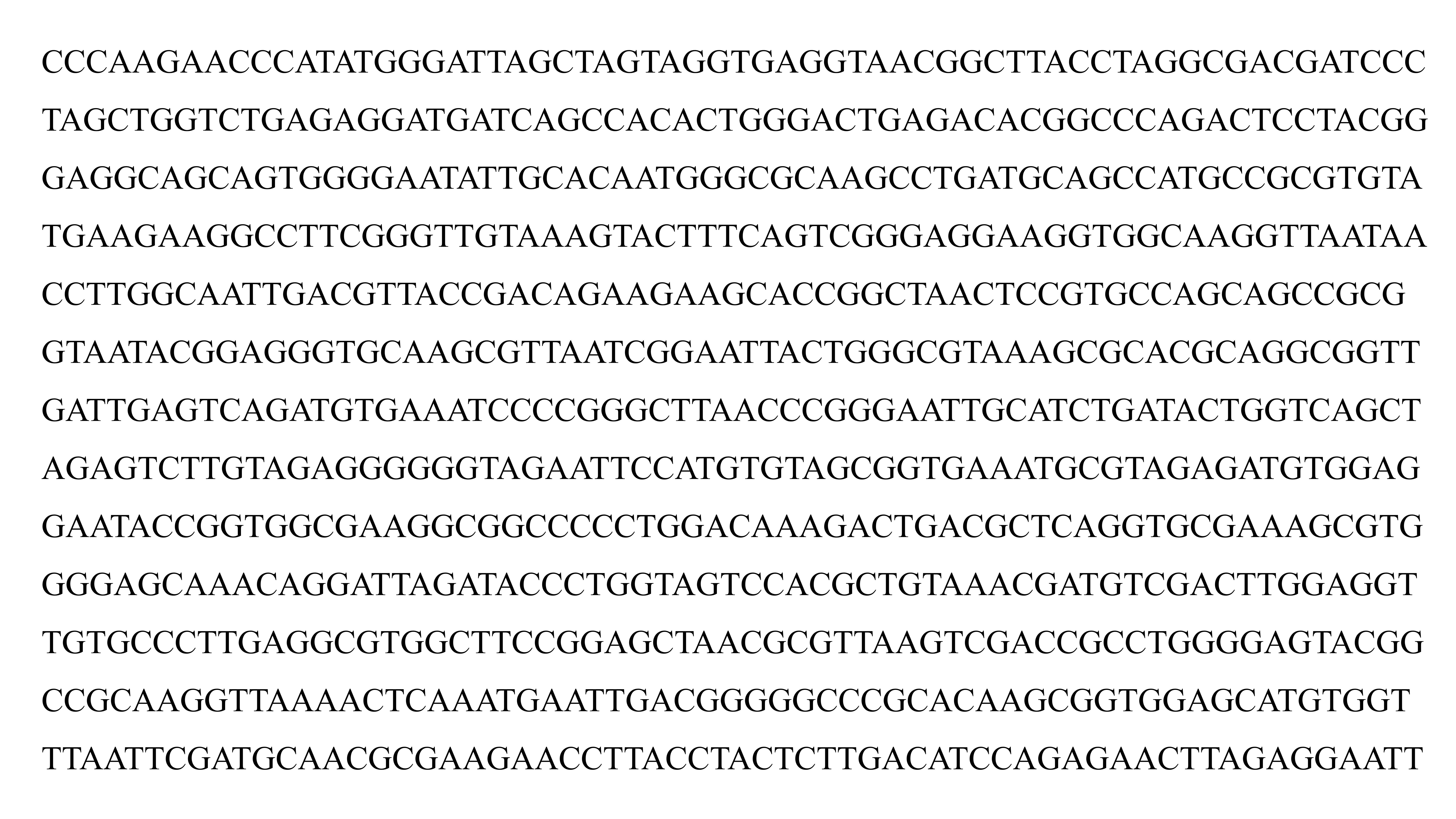

Supplement: SUPPLEMENTARY FIGURE 1 — 16S rRNA gene sequence obtained from strain NM-11 after amplification. [file Image_1.TIF]
